# Supplementary material for: Self-powered ultraflexible photonic skin for continuous bio-signal detection via air-operation-stable polymer light-emitting diodes
Source: Nat Commun. 2021 Apr 14;12:2234. doi: 10.1038/s41467-021-22558-6 (PMC8047008; doi:10.1038/s41467-021-22558-6)
Supplement: Supplementary file 1 — Supplementary Information [file 41467_2021_22558_MOESM1_ESM.pdf]

Self-powered ultraflexible photonic skin for continuous bio-signal detection via air-operation-stable polymer light-emitting diodes

Hiroaki Jinno[1,2†], Tomoyuki Yokota[1], Mari Koizumi[1], Wakako Yukita[1], Masahiko Saito[3], Itaru Osaka[3], Kenjiro Fukuda[2,4], Takao Someya[1,2,4]

[1]Electrical and Electronic Engineering and Information Systems, The University of Tokyo, 7-3-1 Hongo, Bunkyo-ku, Tokyo 113-8656, Japan

[2]Center for Emergent Matter Science, RIKEN, 2-1 Hirosawa, Wako, Saitama 351-0198, Japan

[3]Department of Applied Chemistry, Graduate School of Engineering, Hiroshima University, 1-4-1 Kagamiyama, Higashi-Hiroshima, Hiroshima 739-8527, Japan

[4]Thin-film Device Laboratory, RIKEN, 2-1 Hirosawa, Wako, Saitama 351-0198, Japan

[†]Present address: Nanomaterials Engineering Research Group, ETH zürich, 8093 Zürich, Switzerland

*Correspondence should be sent to someya@ee.t.u-tokyo.ac.jp

Supplementary Figures

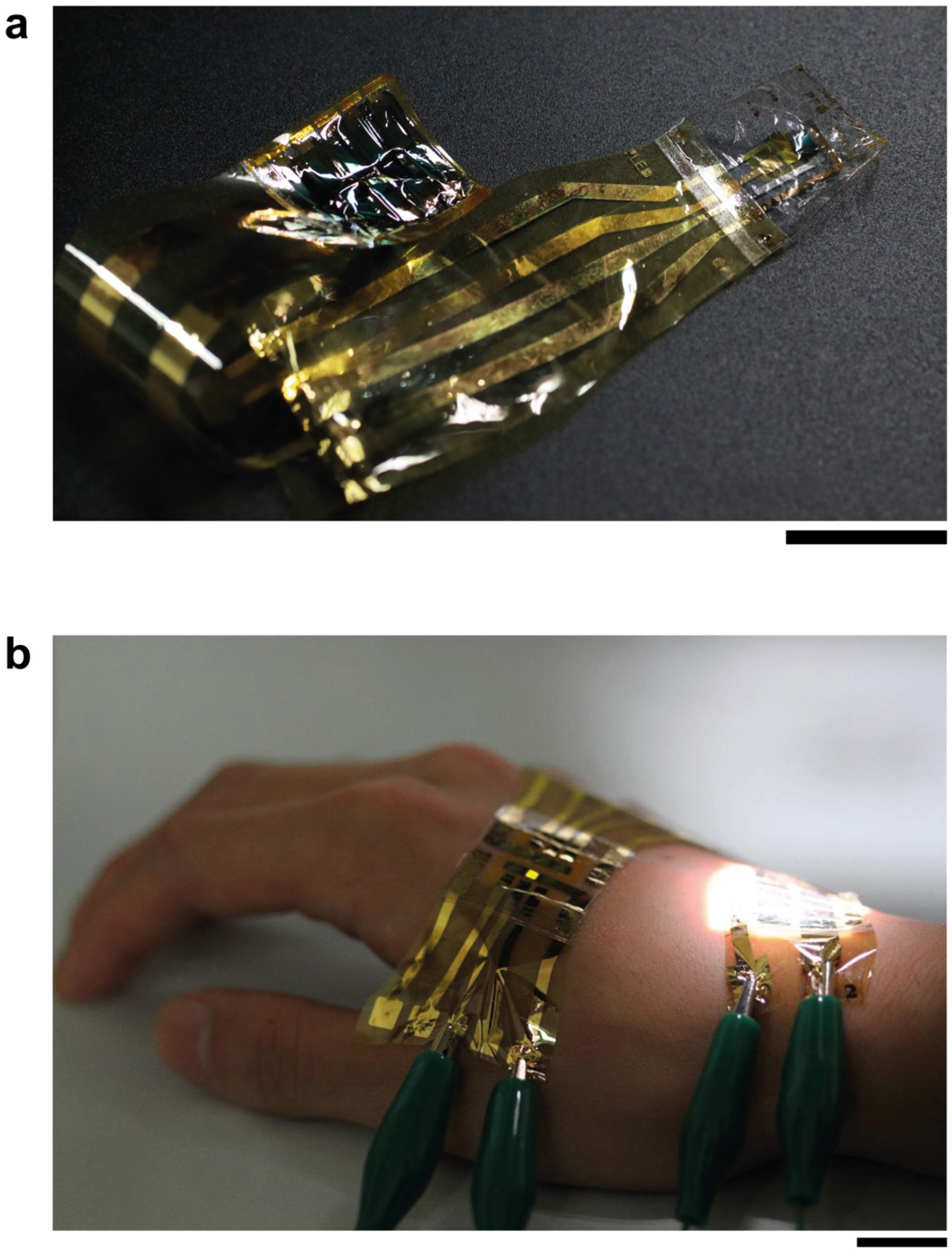

Supplementary Figure 1 | Photographs of Ultraflexible, Self-powered Photoplethysmogram Sensor. a, A photograph of ultraflexible, self-powered photoplethysmogram sensor. Scale bar: 1 cm. **b,** A photograph of an operation of ultraflexible polymer light-emitting diode with 10 series connected ultraflexible organic photovoltaic module powered by one-sun illumination. Scale bar: 1 cm.

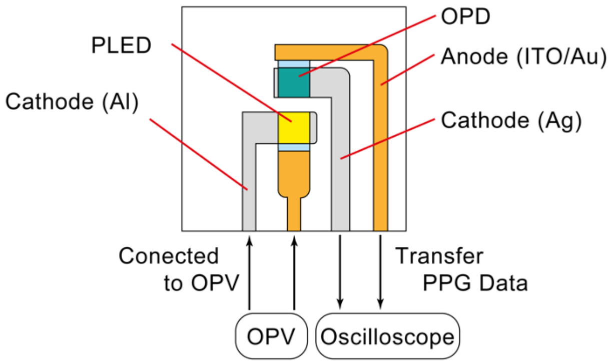

Supplementary Figure 2 | A Top-view Schematic of Photoplethysmogram Sensor. A photoplethysmorgam (PPG) sensor consists of polymer light-emitting diode (PLED) and organic photodiode (OPD). PLED is powered by organic photovoltaic (OPV) module and emits light to fingers. Reflected light from blood vessel is detected by OPD output voltages. The output currents are transferred to oscilloscope and recorded.

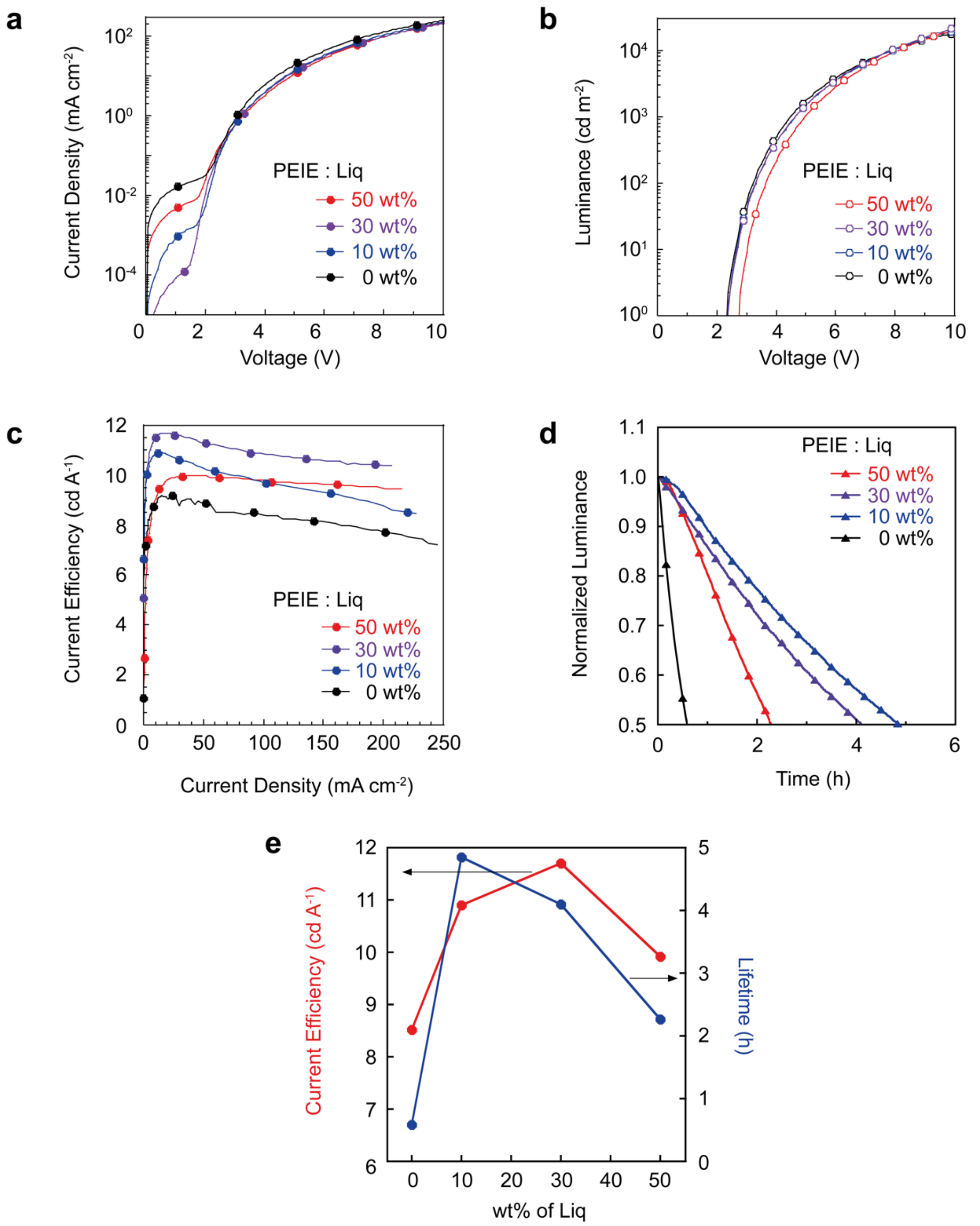

Supplementary Figure 3 | Effect of 8-quinolinolato Lithium wt% on Inverted Polymer Light-Emitting Diodes. a, Current Density-voltage (*J*-*V*) characteristics with 0, 10, 30, 50 wt% doping concentrations of the 8-quinolinolato lithium (Liq) in polyethylenimine ethoxylated (PEIE) layer. **b,** Luminance-voltage (*L*-*V*) characteristics with each doping concentration of Liq **c,** Current Efficiency Characteristics with each doping concentrations of Liq. **d,** Air-operation stabilities of the inverted polymer light-emitting diodes (PLEDs) each doping concentrations of Liq. Operation voltage of each PLED was 8 V. The PLEDs operated under ambient condition (20 ºC, 20% relative humidity). **e,** Summary of the variations in the efficiency and lifetime of the PLED with varying doping concentrations of Liq.

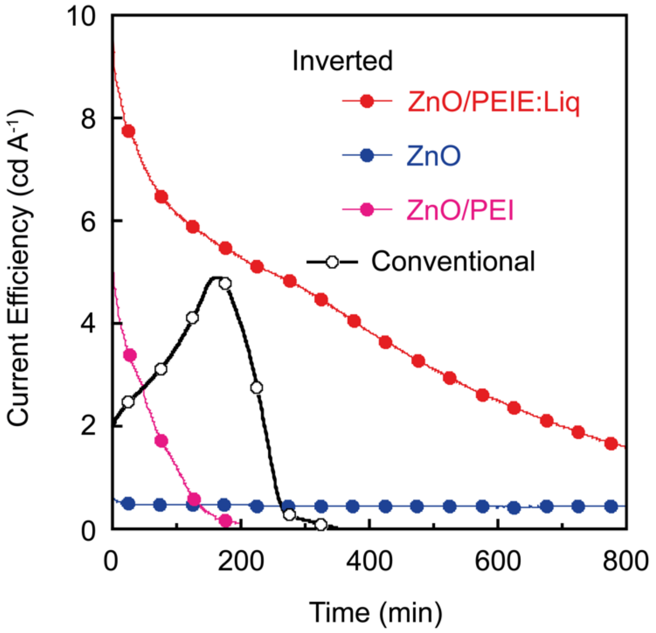

Supplementary Figure 4 | Polymer Light-Emitting Diodes Stability under Constant Voltage Operation in Ambient Air. Air stability between various reported structures and electron transporting layers (ETLs) of polymer light-emitting diode (PLED) were compared. Conventional structure of PLED has a stack of Indium tin oxide (ITO) / Poly(3,4-ethylenedioxythiophene)-poly(styrenesulfonate) (PEDOT:PSS) /Superyellow (SY)/Sodium Fluoride/Al. Those of inverted structures are ITO/Zinc oxide (ZnO) /SY/Molybdenum oxide (MoO_X) /Al, ITO/ZnO/ Polyethylenimine (PEI) /SY/MoO_X/Al, ITO/ZnO/Polyethylenimine ethoxylated (PEIE) :8-quinolinolato lithium (Liq) /SY/MoO_X/Al, respectively. Operation voltage was 8 V. The devices were operated in ambient air condition (20 °C, 20% relative humidity).

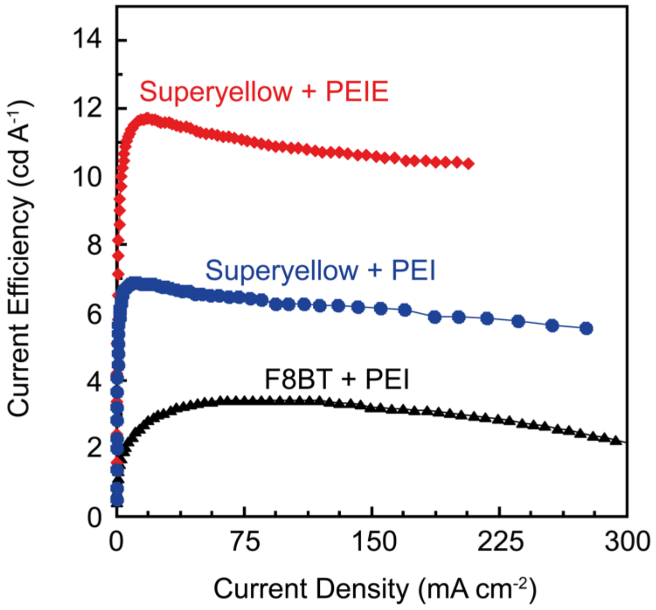

Supplementary Figure 5 | A Comparison of the Current Efficiencies of Various Inverted Structure Polymer Light-Emitting Diodes. The red line indicates current efficiencies of the polymer light-emitting diode (PLED) with a stack of Indium tin oxide (ITO) /Zinc oxide (ZnO) / Polyethylenimine ethoxylated (PEIE) :8-quinolinolato lithium (Liq) /Superyellow (SY) / Molybdenum oxide (MoO_X) /Al. The blue line indicates those with ITO/ZnO/ Polyethylenimine (PEI) /SY/MoO_X/Al and the black line indicates those with ITO/ZnO/PEI/F8BT/MoO_X/Al, respectively.

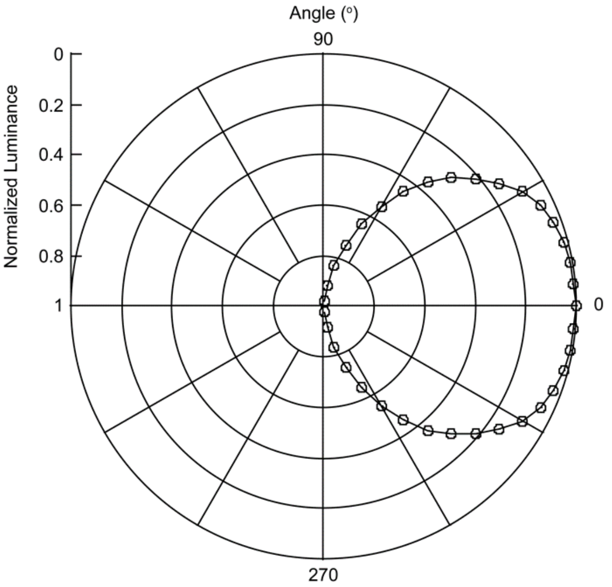

Supplementary Figure 6 | Angular Distribution of the Polymer Light-Emitting Diode (PLED) Luminance. Angular distribution was measured under the operation voltage of 6 V. Initial value of PLED luminance was 2153 cd/m^2 with the angle of 0°.

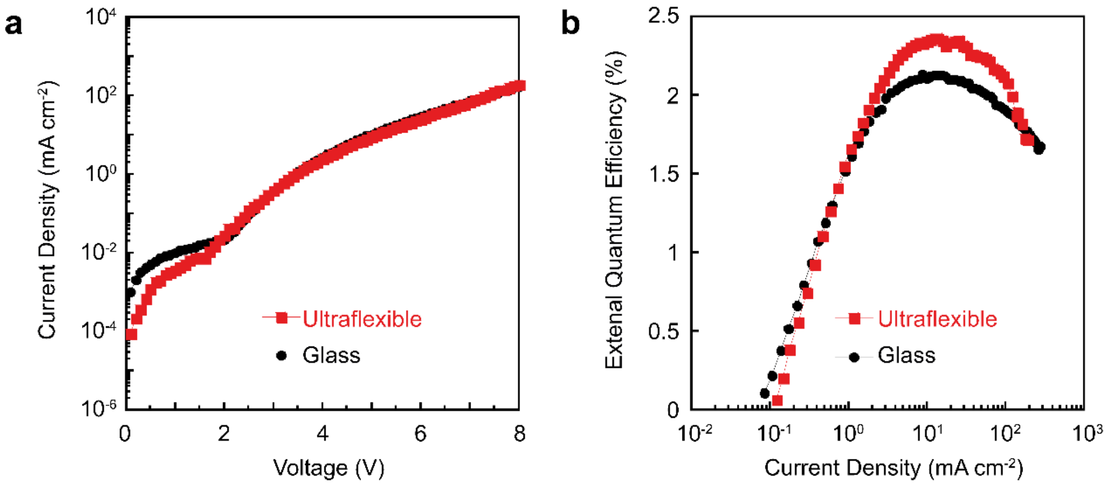

Supplementary Figure 7 | Current Density- Voltage and External Quantum Efficiency (EQE) Characteristics of the Inverted Polymer Light-Emitting Diodes on Different Substrates. The black line and the red line with filled circles indicate current density-voltage (*J*-*V*) characteristics of the inverted polymer light-emitting diodes (PLED) on glass substrate and on ultraflexible substrate, respectively. The black line and red line with blank circles indicate luminance-voltage (*L*-*V*) characteristics of the inverted PLED on glass and on ultraflexible substrate, respectively. Ultraflexible PLEDs were measured in freestanding state without supporting glass substrate. The inverted PLED structure is Indium tin oxide/Zinc oxide/Polyethylenimine/Superyellow/Molybdenum oxide/Au.

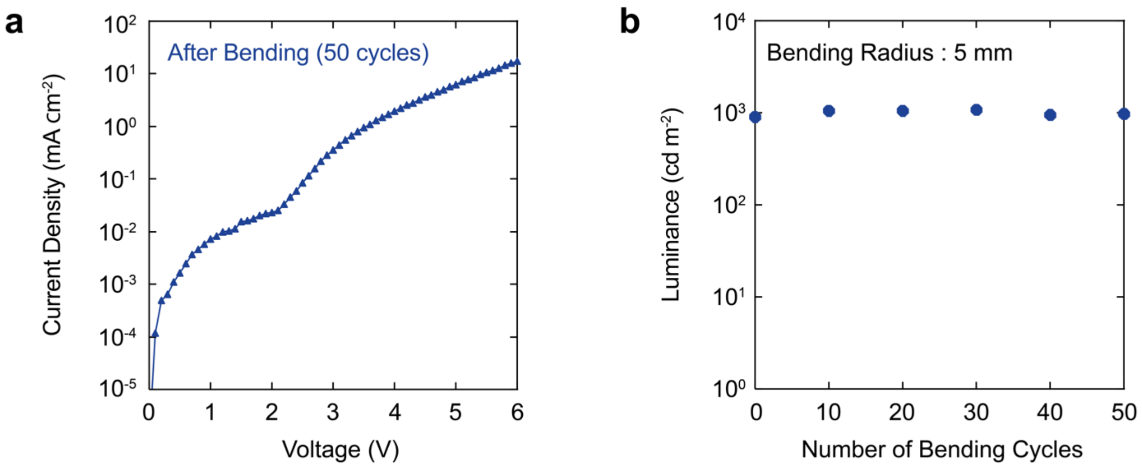

Supplementary Figure 8 | Cyclic Bending Test of Ultraflexible Polymer Light-Emitting Diode (PLED). a, Current density-voltage (*J-V*) curves before and after 50 cycles of bending. **b,** PLED Luminance dependence before and after 50 cyclic bending. The operational voltage applied to PLED was 6 V. Bending radius of 5 mm was applied to the cyclic bending test.

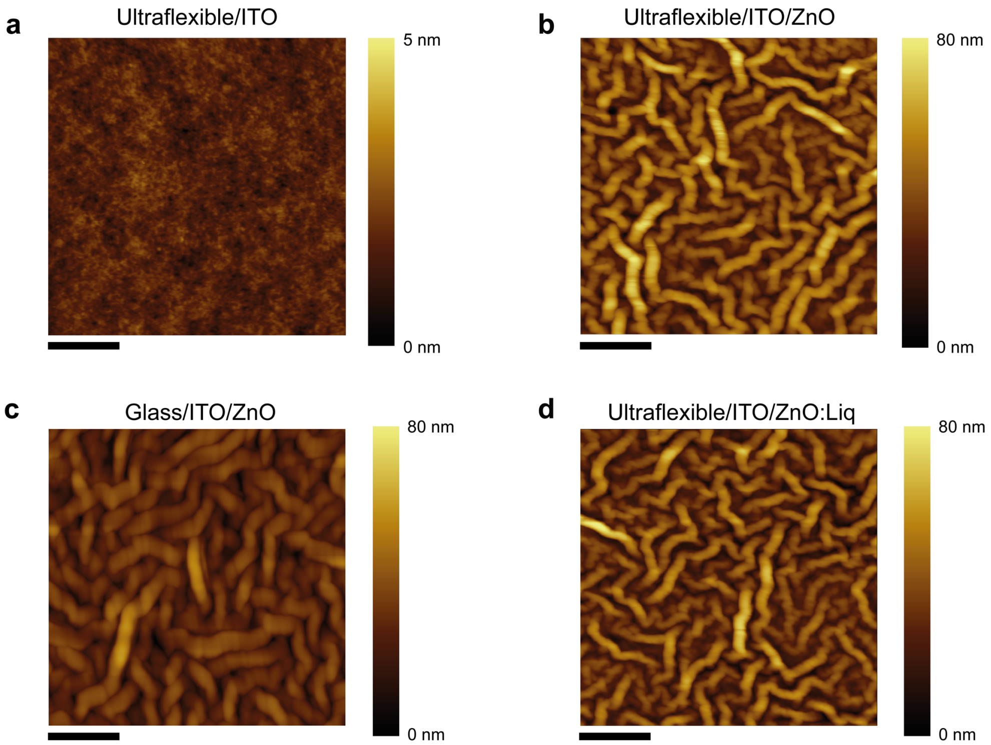

Supplementary Figure 9 | Surface Images of Polymer Light-Emitting Diode (PLED) interface layers with Atomic Force Microscope (AFM). Surface images of **a,** Indium tin oxide (ITO) electrode on top of 1.5 μm-thick ultraflexible substrate, **b,** Zinc oxide (ZnO) interface layer on top of ITO and ultraflexible substrate, **c,** ZnO interface layer on top of ITO and glass substrate, and **d,** ZnO: 8-quinolinolato lithium (Liq) interface on top of ITO and ultraflexible substrate. Root Mean Square of surface roughness of ZnO layer on ultraflexible substrate is 12.5 nm and that of ZnO layer on glass substrate is 7.79 nm, respectively. Black scale bar of each image indicates 1 μm.

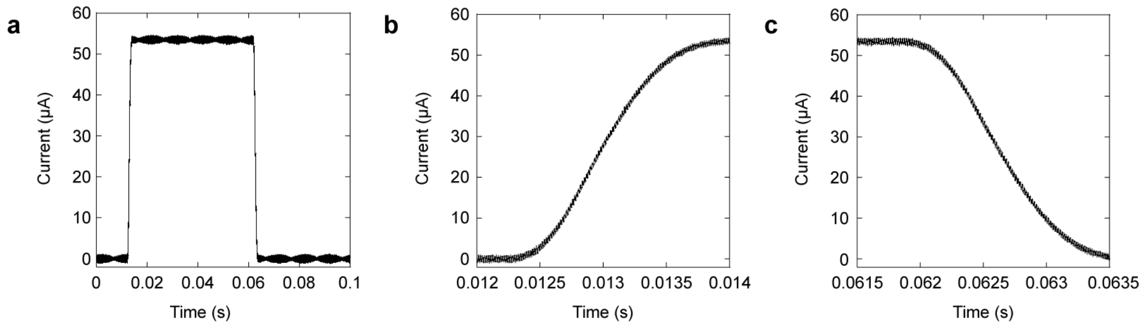

Supplementary Figure 10 | Time Response Measurement of Organic Photodiode (OPD). a, Time response of OPD short-circuit current under light **b,** Magnified Graph of time response measurement for OPD rising time and **c,** falling time. A laser of 550 nm was used as a light source and an optical chopper was operated with 50 Hz frequency. The intensity of the laser light source was measured as 8 mW/cm^2 at a wavelength of 550 nm with an optical power meter (1936-R, Newport).

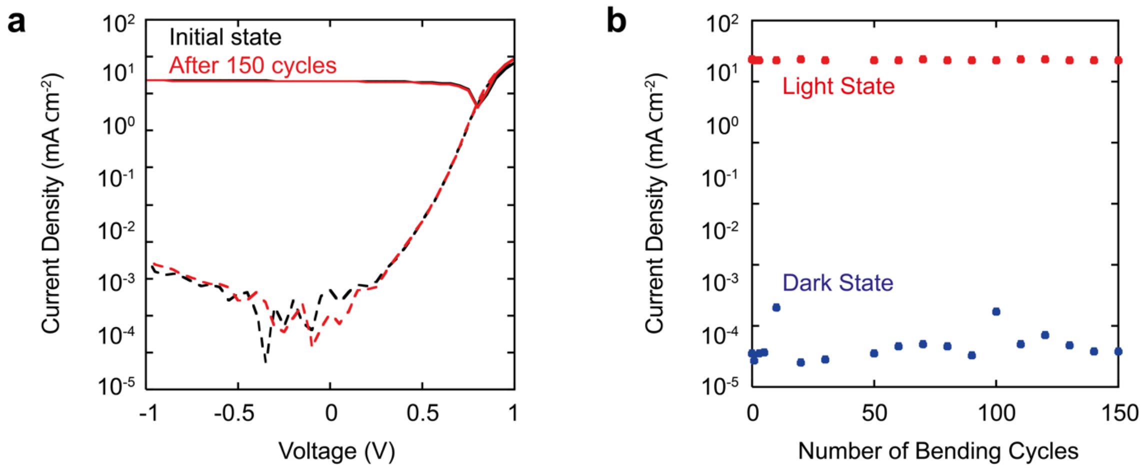

Supplementary Figure 11 | Cyclic Bending Test of Ultraflexible Organic Photodiode (OPD). a, Current density-voltage (*J*-*V*) curves before and after 150 cycles of bending. **b,** Short-circuit current (J_{SC}) of OPD under light condition and dark condition before and after 150 cyclic bending. In the light condition, ultraflexible OPD was irradiated with ambient room light. Bending radius of 5 mm was applied to the cyclic bending test.

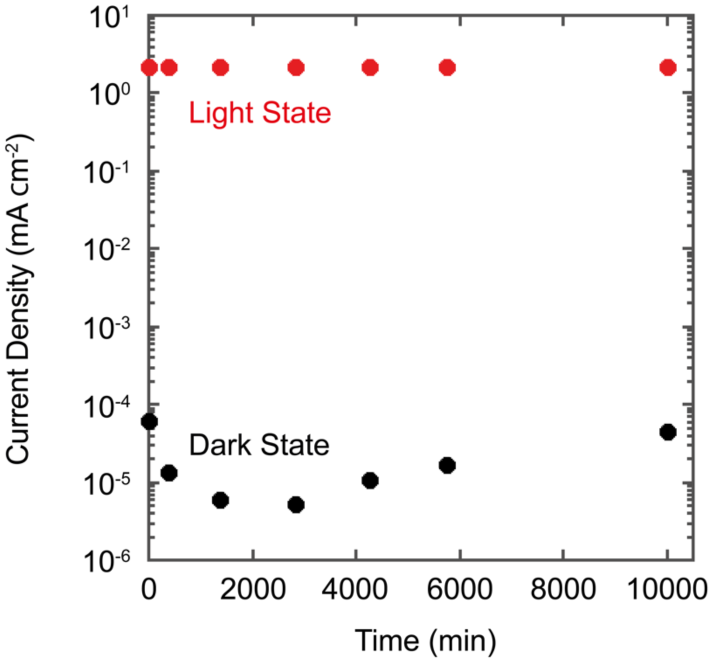

Supplementary Figure 12 | Storage Stability of Ultraflexible Photodiode (OPD) Under Ambient Air. The devices were stored under ambient air (Room temperature, 30% relative humidity, approximately) and dark condition. The photocurrent of OPD in light state were measured with 550 nm laser irradiation.

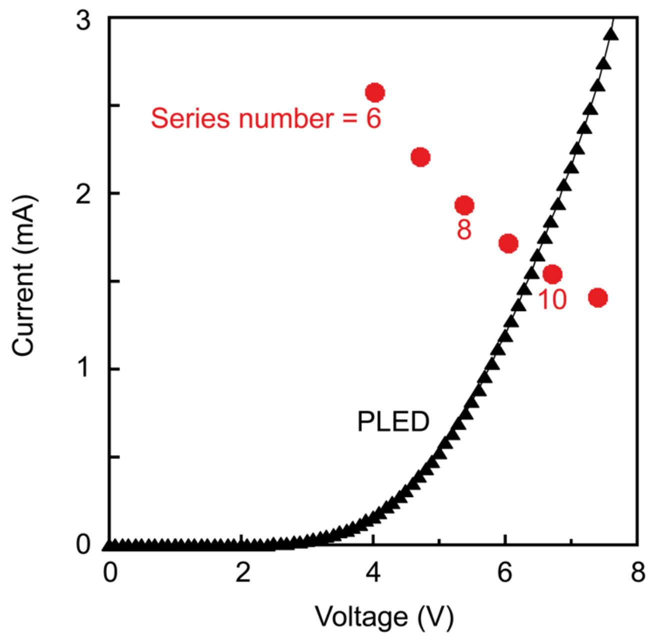

Supplementary Figure 13 | Calculated Output Currents and Voltages of Organic Photovoltaic Modules with Various Numbers of Series Connection. Output currents and voltages of organic photovoltaic (OPV) modules were overlaid with the *I*-*V* curve of polymer light-emitting diode (PLED). As the increase in the number of series connections, maximum power points of OPV module were shifted to lower current and higher voltage region.

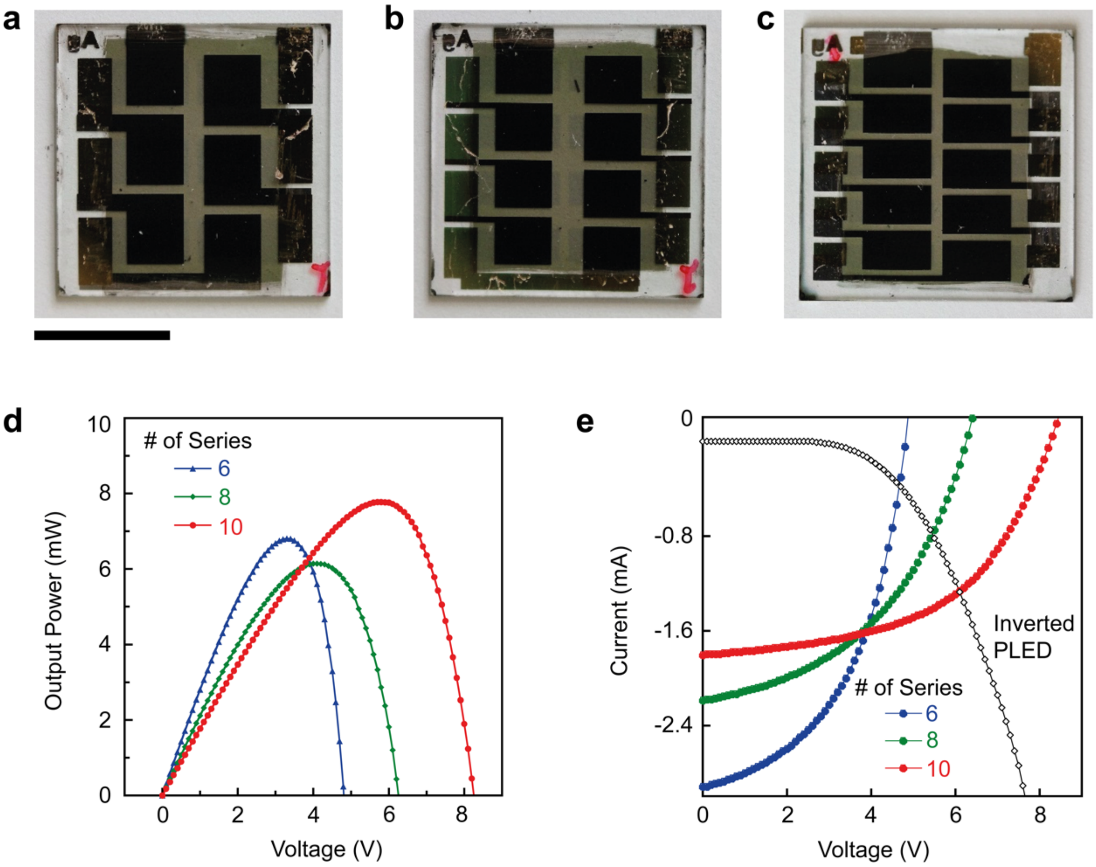

Supplementary Figure 14 | Organic Photovoltaic Modules with Series Numbers of 6, 8, 10. Top-view photographs of **a,** 6 series **b,** 8 series **c,** 10 series connection organic photovoltaic (OPV) module. **d,** Output power characteristics of OPV modules with 6, 8, and 10 series connection **e,** Current-voltage characteristics of OPV modules with 6, 8, 10 series connection. OPV *I-V* curves were overlaid with the reversed *I-V* curve of polymer light-emitting diode (PLED).

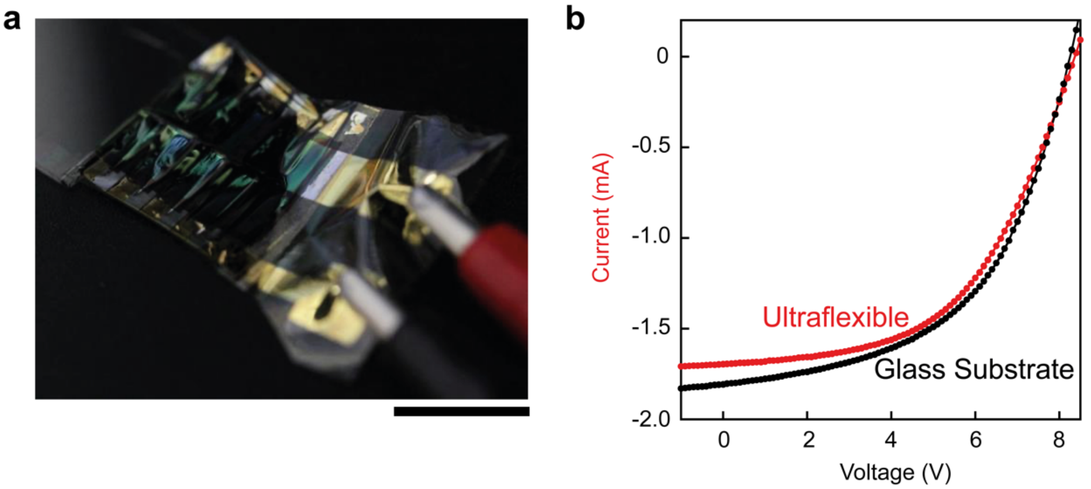

Supplementary Figure 15 | Organic Photovoltaic Modules with 10 Series Connection. **a,** A photograph of an ultraflexible organic photovoltaic (OPV) module. Scale bar indicates 1 cm. **b,** Current-voltage (*I*-*V*) characteristics of OPV modules on glass substrate and on ultraflexible substrate. Black line indicates a *I*-*V* curve of OPV module on glass substrate and red line indicates that on ultraflexible substrate.

Supplementary Table 1 | Photovoltaic Characteristics of Organic Photovoltaic Modules.

	on Glass	on Ultraflexible
J_{SC} (mA cm^{-2})	15.0	14.0
V_{OC} (V)	0.83	0.82
FF	0.52	0.51
PCE (%)	6.5	5.8

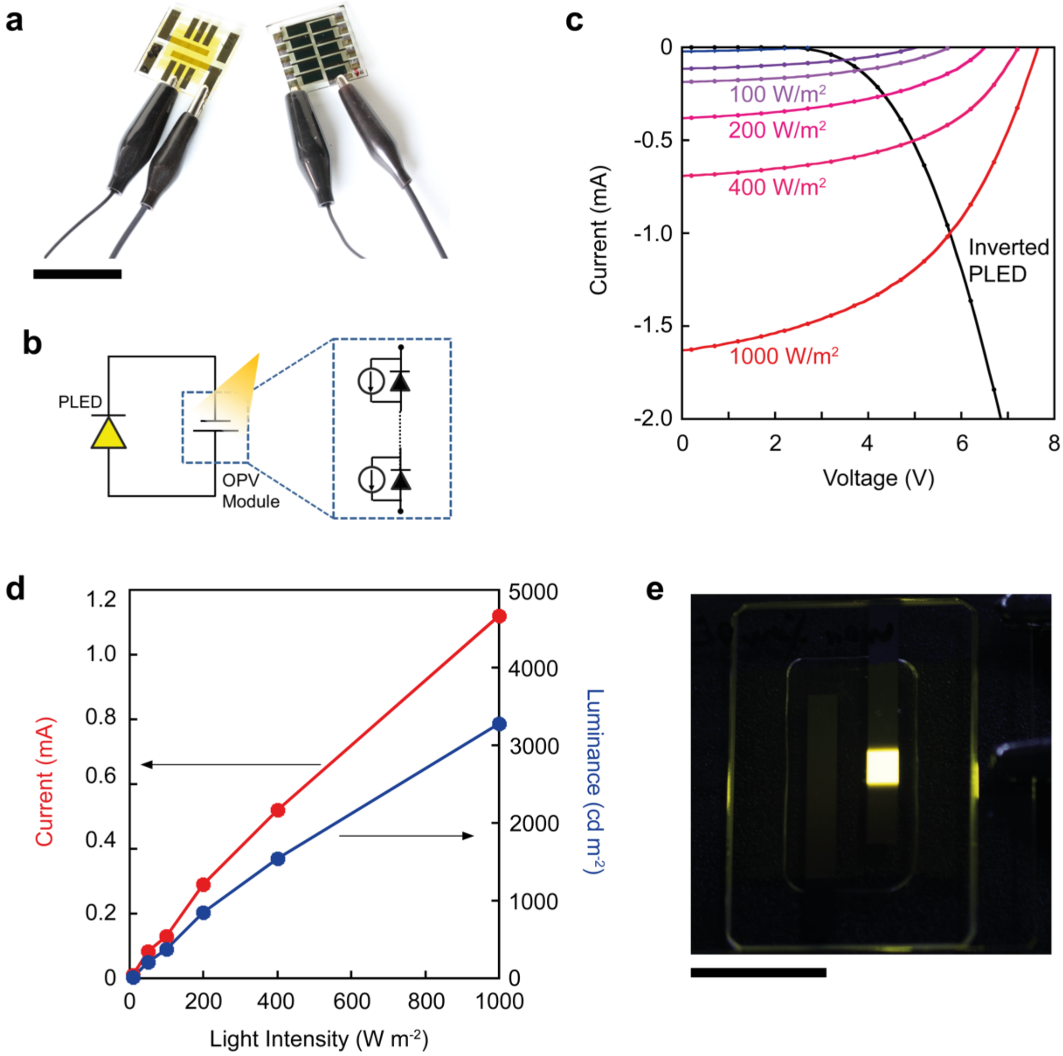

Supplementary Figure 16 | Polymer Light-Emitting Diode Operation with a 10 Series Connected Organic Photovoltaics Module. a, A photograph of polymer light-emitting diode (PLED) operation with the organic photovoltaic (OPV) module. Scale bar indicates 1 cm. **b,** A schematic of the electrical circuit with the PLED and OPV module. **c,** Light intensity dependences of current voltage (*I-V*) characteristics of the OPV module. OPV *I-V* curves were overlaid with the PLED *I-V* curve. **d,** Dependences of circuit current and PLED luminance on the light intensity of input sunlight. Red line indicates the circuit currents and blue line indicates the PLED luminance. **e,** A top-view photograph of the PLED operation in 50W/m^2 illumination of simulated sunlight. Scale bar indicates 1 cm.

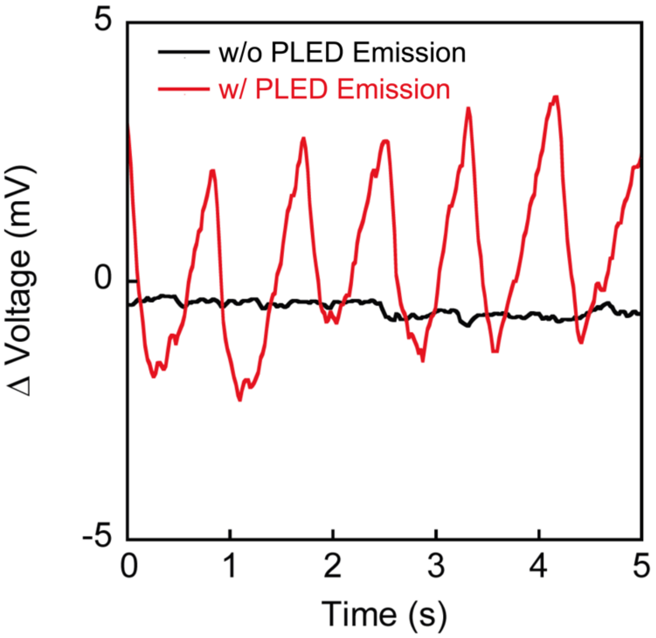

Supplementary Figure 17 | Output Voltages of Organic Photodiode with Switching on and off Polymer Light-Emitting Diode. Polymer light-emitting diode (PLED) light was switched on and off by intermittently exposing the OPV module to simulated sunlight.

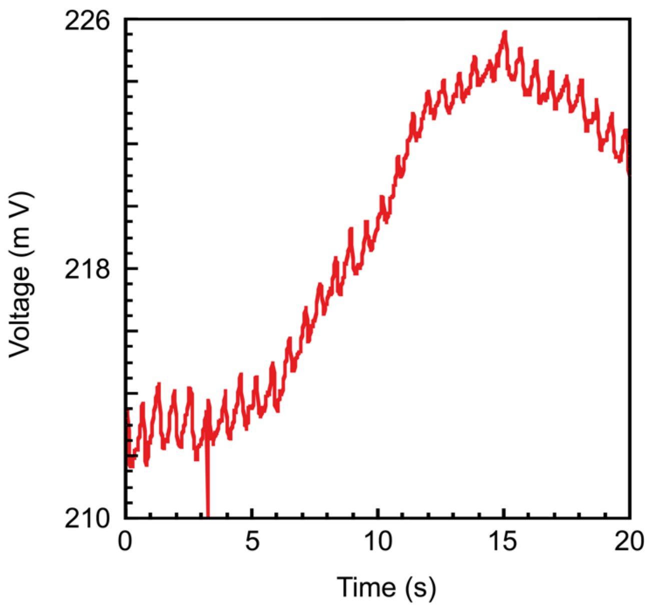

Supplementary Figure 18 | Long-term Measurement of Photoplethysmogram (PPG) Systems on Ultraflexible Substrate. Ultraflexible polymer light-emitting diode (PLED) was powered by ultraflexible organic photovoltaic module. the voltage was obtained from ultraflexible orgaic photodiode with the PLED illumination. All devices were used with the supporting glass substrate.

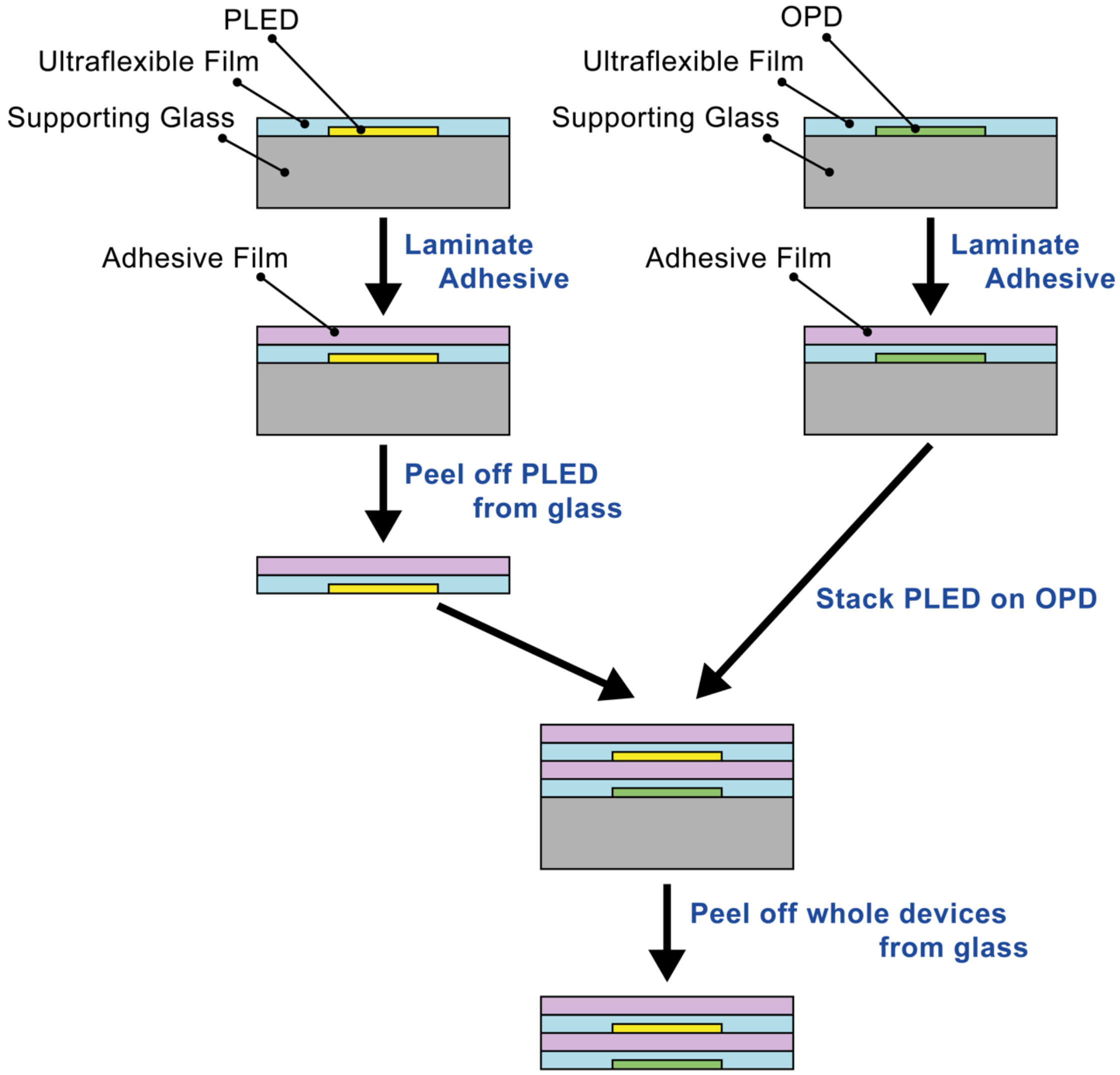

Supplementary Figure 19 | Schematics of Lamination Method with Adhesive Tape to Combine Ultraflexible, Self-powered Photoplethysmogram Sensor. First, an ultraflexible polymer light-emitting diode (PLED) was peeled off from a glass supporting substrate with an adhesive tape (referred as freestanding state). Consequently, the ultraflexible PLED with freestanding state was laminated on an ultraflexible organic photodiode (OPD) with another adhesive tape to form a stack. At last, the ultraflexible PLED and the ultraflexible OPD were peeled off from a glass supporting substrate again to combine an ultraflexible, self-powered photoplethysmogram sensor.

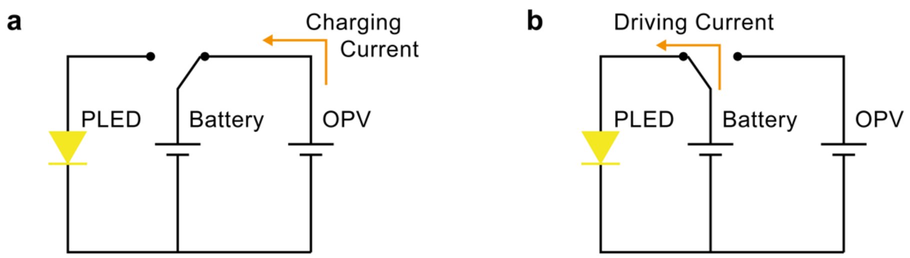

Supplementary Figure 20 | Schematics of Electrical Circuit with Self-powered Device combined with Rechargeable Battery. a, Electrical Circuit Schematics during the circuit operates as photo-charging mode, which the rechargeable battery will be charged by the photo-current generated by organic photovoltaic (OPV) module. **b,** Electrical Circuit Schematics during the circuit operates as driving mode, which the charged battery will drive the polymer light-emitting diode (PLED) with a constant voltage and current to get a stable operation.

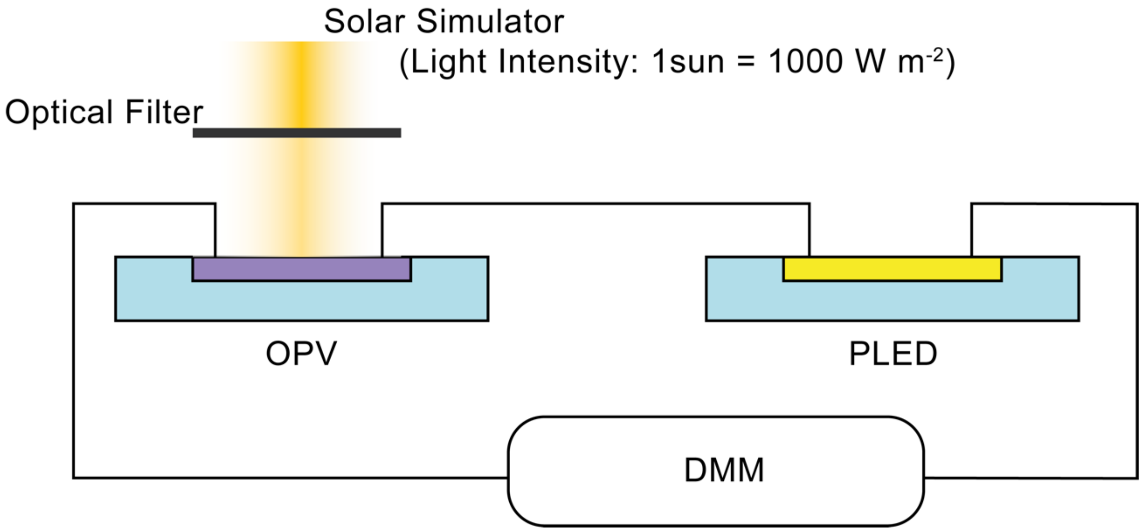

Supplementary Figure 21 | A Schematic of the Experiment Setup of Polymer Light-Emitting Diode (PLED) Operation with Organic Photovoltaic (OPV) Module as a power source.
